# Whole genome sequencing resolves 10 years diagnostic odyssey in familiar myxoma

**DOI:** 10.1038/s41598-023-41878-9

**Published:** 2023-09-05

**Authors:** Sára Pálla, Judit Tőke, Anikó Bozsik, Henriett Butz, János Papp, István Likó, Enikő Kuroli, András Bánvölgyi, Mátyás Hamar, Jerome Bertherat, Márta Medvecz, Attila Patócs

**Affiliations:** 1 Department of Dermatology, Venereology and Dermatooncology, Semmelweis University, Budapest, Hungary; 2 Department of Internal Medicine and Oncology, Semmelweis University, Budapest, Hungary; 3 ENDO-ERN HCP Semmelweis University, Budapest, Hungary; 4 Department of Molecular Genetics, National Institute of Oncology, Ráth György U. 7-9, 1122 Budapest, Hungary; 5 Hereditary Cancers Research Group, Eötvös Loránd Research Network, Semmelweis University, Budapest, Hungary; 6 National Tumorbiology Laboratory, Budapest, Hungary; 7 Department of Surgery, Transplantation and Gastroenterology, Semmelweis University, Budapest, Hungary; 8 Université Paris Cité, Institut Cochin, Inserm U1016, Paris, France; 9 Department of Endocrinology and National Reference Center for Rare Adrenal Disorders, Hôpital Cochin, Assistance Publique Hôpitaux de Paris, Paris, France; 10 ERN-Skin Semmelweis University, Budapest, Hungary; 11 National Institute of Oncology, Oncology Biobank Center, Budapest, Hungary; 12 Department of Laboratory Medicine, Semmelweis University, Budapest, Hungary; 13 Department of Pathology and Experimental Cancer Research, Semmelweis University, Budapest, Hungary

**Keywords:** Disease genetics, Medical genetics, Next-generation sequencing

## Abstract

Carney complex (CNC) is an ultrarare disorder causing cutaneous and cardiac myxomas, primary pigmented nodular adrenocortical disease, hypophyseal adenoma, and gonadal tumours. Genetic alterations are often missed under routine genetic testing. Pathogenic variants in *PRKAR1A* are identified in most cases, while large exonic or chromosomal deletions have only been reported in a few cases. Our aim was to identify the causal genetic alteration in our kindred with a clinical diagnosis of CNC and prove its pathogenic role by functional investigation. Targeted testing of *PRKAR1A* gene, whole exome and whole genome sequencing (WGS) were performed in the proband, one clinically affected and one unaffected relative. WGS identified a novel, large, 10,662 bp (10.6 kbp; LRG_514t1:c.-10403_-7 + 265del; hg19, chr17:g.66498293_66508954del) deletion in the promoter of *PRKAR1A* in heterozygous form in the affected family members. The exact breakpoints and the increased enzyme activity in deletion carriers compared to wild type carrier were proved. Segregation analysis and functional evaluation of PKA activity confirmed the pathogenic role of this alteration. A novel deletion upstream of the *PRKAR1A* gene was proved to be the cause of CNC. Our study underlines the need for WGS in molecular genetic testing of patients with monogenic disorders where conventional genetic analysis fails.

## Introduction

Carney complex (CNC) is an ultrarare, multiorgan disease. Its typical manifestations include myxomas (cardiac − mostly atrial − and cutaneous), other benign dermatological lesions and endocrine tumours (including primary pigmented nodular adrenal hyperplasia and tumours affecting the gonads)^1,2^. Clinical diagnosis of CNC can be made using the diagnostic criteria and supplemental criteria for CNC summarised by Stratakis et al.^3^.

Carney complex is caused by a pathogenic variant of the gene encoding the 1A regulatory subunit of protein kinase A (*PRKAR1A,* OMIM accession number: 188830)^4^. Disease-causing mutations can be detected at any site of the gene but most of them map to exonic and splice sites^5^. Importantly, there is no mutation hotspot, almost every family has its own pathogenic variant. Significant genotype–phenotype associations have not been observed, however some studies reported that certain mutations in *PRKAR1A* gene or sequence variants in other genes (i.e. *PDE11A*) may lead to the development of adrenal and/or testicular tumours^6,7^. Clinical data and genetic results of the largest CNC patient cohort (n = 353) to date revealed some important and significant observations. From the total of 353 CNC patients from 185 families, 113 patients (32%) were classified as sporadic cases, while the remaining 240 (68%) patients had familial disorder. The most frequent tumour was primary pigmented nodular adrenocortical disease (PPNAD) diagnosed in 212 patients (60%), which was followed by testicular (41% of males), thyroid (25%), ovarian (14% of females) and pituitary (12%) neoplasias. From the nonendocrine components lentiginosis was observed most frequently (in 248 of 353 patients (70%)). Pathogenic and likely pathogenic (P/LP) variants in the *PRKAR1A* gene could be identified in up to 80% of the cases with familial presentation, while large exonic or chromosomal deletion involving the *PRKAR1A* locus have been reported only in a few cases^8^. Identification of these later genetic alterations is very often missed under routine genetic testing where only coding and splice regions are tested by DNA sequencing. Nowadays, whole exome and whole genome sequencing (WES and WGS) are powerful tools for comprehensive genetic testing, however, in well-defined monogenic syndromes, the first choice for genetic analysis is the evaluation of the suspected gene. Large-scale genetic testing (WES and WGS) is also available, but variants located in intergenic or noncoding regions are frequently not reported^9^.

P/LP variants of the *PRKAR1A* gene result in functional loss of the PRKAR1A protein^10^. Loss of inhibitory function of the PRKAR1A results in overactivation of the protein kinase A (PKA) signalling. The activity of PKA is known to be induced by the cyclic AMP produced by G-protein-coupled hormone receptors such as follicle-stimulating hormone, adrenocorticotrophic hormone, thyroid-stimulating hormone, and melanocyte-stimulating hormone. The endocrinologic symptoms in CNC reflect excessive signalling by these hormones provoked by loss of function of PKARIα as the suppressor of PKA.

PKA protein plays a role in the regulation of carbohydrate and lipid metabolism, and it is one of the components of the signal transmission route related to guanine nucleotide binding protein coupled receptors (GPCRs). This enzyme is made up of two regulatory and two catalytic subunits. Cyclic adenosine monophosphate (cAMP) connects to the regulatory subunits, and afterwards, the released catalytic subunits phosphorylase different target proteins. Besides ion channels and other enzymes, PKA activates the transcription factor: cAMP response element-binding (CREB) protein. The *PRKAR1A* gene encodes one of the regulatory subunits of this important enzyme.

To date, only symptomatic management of CNC is available. The repeating surgical removal of the cutaneous tumours can restore normal anatomy and function, therefore improving significantly the patients’ Quality of Life. After the surgical removal of the cardiac myxoma, there is a significant risk for recurrence as it has been observed in 12–22% of all cases^11,12^. Once the typical clinical signs are identified genetic testing is indicated. For clinical follow up a multidisciplinary team is required to adequately screen and approach further elements of CNC that are more likely to express later in life^13^.

In this report, we describe a family presenting with familial multiple myxomas which indicated CNC. Endocrine abnormalities were very mild and routine genetic testing performed by targeted analysis of the *PRKAR1A* gene followed by WES failed to identify any P/LP *PRKAR1A* variant. WGS by identifying a large promoter deletion ended our 10-year diagnostic odyssey in this pedigree with a clear clinical diagnosis of CNC. Functional assay proved that this deletion results in haploinsufficiency and loss of inhibitory effect on PKA activity.

## Methods

### Case report

#### Dermatological findings

**Figure 1:**
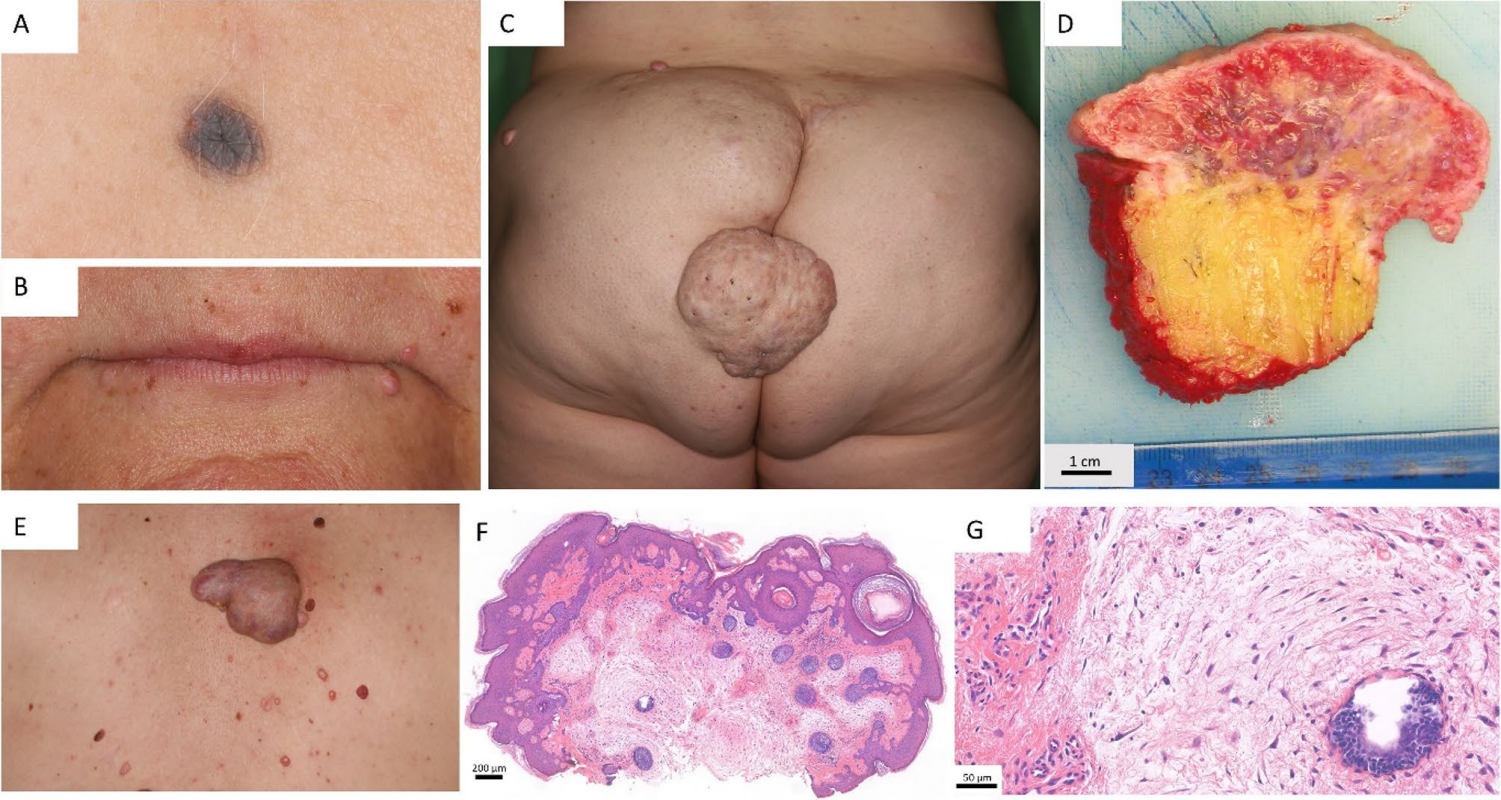
Dermatological manifestations in proband. Naevus coeruleus (**A**). Perioral lentiginosis (**B**). Multiple subcutaneous and a large, exophytic 8 × 8 cm cutaneous myxoma on the left gluteal region (**C**), and cross section of the removed exophytic tumour (**D**). Multiple cutaneous myxomas on the back (**E**) and hematoxylin and eosin stained tissue sections with original magnification × 45 (**F**) and × 286 (**G**). Histopathology identified myxoid tumour tissue infiltrating subcutaneous connective and adipose tissues. Elongated, monomorphic cells were present in myxoid substance of tumour foci, without cell atypia or proliferative activity.

Starting from the age of 17 years, multiple cutaneous myxomas and left atrial myxoma were observed and surgically treated. At the time of the first dermatologic investigation (at 38 years), blue naevus (Fig. 1a), lentiginosis on the face (Fig. 1b) and cutaneous myxomas (Fig. 1c) raised the possibility of CNC. Numerous (n > 50) cutaneous, subcutaneous, and mucosal; sessile, and pedunculated pink lesions, with a diameter size ranging from 0.5 cm to 15 cm were observed (Figs. 1c,d,e). Cutaneous ultrasonography was performed on large subcutaneous tumours describing well-defined hypoechoic masses in the hypodermis with hypervascularisation in the marginal areas. Histopathological examination described hypocellular myxoid neoplasia that confirmed the diagnosis of cutaneous myxomas (Figs. 1f,g). The myxomas appeared in the axial region (head and neck; thoracic, abdominal, dorsal, lumbar, gluteal, and genital areas), sparing the extremities. The presence of myxomas in specific areas, such as the ear, external ear canals, upper eyelid, and areola mammae was observed. Hence the disfiguring lesions, the patient required multiple excisional cutaneous surgeries.

Lentiginosis of the face was also observed, affecting the centrofacial, perioral, and periocular areas (Fig. 1b). Lentigines were present on the eyelids, canthi, irises, lips, and ears, and were not associated with exposure to light. Our patient had a single blue naevus confirmed by dermoscopy (Fig. 1a).

Other non-specific skin lesions: fibrous papule on the nose, café-au-lait macules (n = 2), histologically confirmed intradermal naevi (n = 4), pigmented naevi (n < 25), dermatofibroma, haemangiomas, atrophic scars on the knee, keratosis pilaris on the arms, and multiple epidermoid cysts and open comedones on the trunk were also observed.

Based on the findings a clinical diagnosis of Carney complex has been made and endocrinological investigations were initiated.

#### Endocrinological findings

At the age of 39, she was diagnosed with polycystic right ovarian morphology and hepatic steatosis (elevated liver enzymes: AST, 121 U/L (Reference range -RR: < 40 U/L); ALT, 76 U/L (RR: < 40 U/L); gGT: 53 U/L, (RR: < 40 U/L). Immunoserology for viral and autoimmune hepatitis was negative. At the age of 41 years, she was diagnosed with mild hypertension, which was well controlled with nebivolol (5 mg q.d.). She reached the menarche at the age of 11; her periods were regular. She gave birth to two children.

At the time of the first endocrinological examination, the patient was 41 years old. At this time, she was presented with 40 kg weight gain over 17 years, and her body mass index was 42.3 kg/m^2^.

Midnight salivary cortisol (3.22 nmol/L; RR: < 11.90 nmol/L), morning serum cortisol (447.3 nmol/L; RR: 220–690 nmol/L) and 24-h urinary free cortisol (279.8 and 332.0 nmol; RR: 100.0–379.0 nmol/L) were within normal ranges. However, she had elevated midnight serum cortisol (304.30 nmol/L; RR: < 136.00 nmol/L) and non-suppressible serum cortisol of 65.45 nmol/L and 54.91 nmol/L following 1 mg and 8 mg dexamethasone overnight suppression tests, respectively. Serum adrenocorticotropic hormone (ACTH) concentration (23 pg/mL; RR: 5–60 pg/mL) and morning serum dehydroepiandrosterone sulphate (4.16 µmol/L; RR: 1.65–11.0 µmol/L) were within the reference ranges. HOMA-index was slightly elevated (5.24; RR: 0.00–4.40) and serum osteocalcin level was close to the lower limit of the normal range (12.3 ng/mL; RR:12.0–41.0 ng/mL). Normal values were detected for serum thyroid-stimulating hormone, prolactin, insulin-like growth factor 1, and collagen-crosslinks. The oral glucose tolerance test did not reveal carbohydrate disorder (Table 1). Based on these laboratory results mild autonomous cortisol hypersecretion was concluded. Computed tomography scan of the abdomen did not reveal any visible adrenal mass (data not shown), primary pigmented nodular adrenal hyperplasia (PPNAD) could not be ruled out or confirmed because our patient has not undergone adrenal surgery.

**Table 1:** Hormone levels of the patient with Carney complex.

Hormone	Value at age of 41 years	Values after 18 months of metformin treatment	Reference range
TSH (mU/L)	1.240	0.757	0.35 0–4.940
Midnight salivary cortisol (nmol/L)	3.220	ND	< 11.900
Urinary free cortisol (nmol/24 h)	279.8 and 332.0	ND	100.0–379.0
ACTH (pg/mL)	23	30	5–60
Serum cortisol (nmol/L) 08.00 am	447.30	389.3	220–690
Serum cortisol (nmol/L) 24.00 pm	304.30	ND	< 136
Serum cortisol (nmol/L) following administration of			
1 mg dexamethasone	65.450	65.180	< 50
8 mg dexamethasone	54.910	ND	< 50
DHEA-S (umol/L)	4.16	ND	1.65–11.00
Serum prolactin (ng/mL)	11.08	7.95	1.39–24.20
IGF-1 (ng/mL)	85.4	179.5	50.0–175.0
Serum ß-crosslaps (pg/mL)	333	226	100–573
Serum osteocalcin (ng/mL)	12.30	12.97	12.00–41.00
HOMA_insulin (uU/mL)	24.72	4.79	2.60–24.90
HOMA_glucose (mmol/L)	4.77	4.50	3.00–5.50
HOMA_index	5.24	0.96	0.00–4.40
Oral glucose load, serum glucose 0 min (mmol/L)	5.4	4.9	
Oral glucose load, serum glucose 120 min (mmol/L)	7.2	5.4	

*TSH* thyroid stimulating hormone, *ACTH* adrenocorticotropic hormone, *DHEA-S* dehydroepiandrosterone-sulphate, *GH* growth hormone, *IGF-I* insulin-like growth factor 1.

This study was performed in line with the principles of the Declaration of Helsinki. Informed consent to publish identifying information and images was obtained from the participant.

### Genetic analysis

#### Germline *PRKAR1A* testing by bidirectional Sanger sequencing

Germline genetic analysis of the proband and family members was done following informed consent based on the Ethical approval by the Scientific and Research Committee of the Medical Research Council of the Ministry of Health, Hungary (ETT-TUKEB 4457/2012/EKU), and all methods were performed in accordance with the relevant guidelines and regulations.

DNA was isolated from peripheral blood, and polymerase chain reaction (PCR) followed by bidirectional DNA sequencing was used to test the *PRKAR1A* gene as previously described^14^.

#### Whole exome sequencing (WES) and Whole genome sequencing (WGS)

Library preparation for WES was conducted using DNA Prep with Enrichment (Illumina). Briefly, 100 ng genomic DNA was tagmented, amplified and purified. The exome capture was performed using Illumina Exome Panel probes (Illumina).

WGS library for Illumina sequencing was prepared using NEBNext Ultra II FS DNA Library Prep Kit for Illumina (NEB) following the standard protocol. Briefly, 400 ng genomic DNA was fragmented, end-repaired, and adapter-ligated. Magnetic beads size selection was performed to select 250–300 bp insert size fragments. Finally, the library was amplified according to the manufacturer’s instructions. The quality of the library was checked on 4200 TapeStation System using D1000 Screen Tape (Agilent Technologies), and the quantity was measured on Qubit 3.0 (Thermo Fisher).

Both sequencing runs were done on NovaSeq 6000 instrument (Illumina) with 2 × 150 run paired-end reads.

#### Bioinformatical analysis of WES and WGS data

Sequencing data processing was performed following the Genome Analysis Toolkit (GATK) best practices guidelines as follows: paired-end sequencing data were obtained in FASTQ file format and reads were trimmed using cutadapt to remove adapters and bases where the PHRED quality value was less than 20^15^.

The trimmed reads were aligned to Genome Reference Consortium Human Build hg19 using Burrows‐Wheeler Aligner (bwa-mem2-2.0)^16^. Picard tools were used to sort, MarkDuplicates and index reads^17^. Base Quality Score Recalibration (BQSR) was performed using GATK^9^. Variant discovery was performed in two steps: (1) single‐sample variant calling was performed using HaplotypeCaller in GATK, (2) followed by GenotypeGVCFs to combine variants from single‐sample gVCFs to the multisample VCF. Variant annotation was predicted using Funcotator.

Copy number variations (CNVs) were called by counting target read counts across individual genomes and comparing the ratios between patients and healthy individuals. The uniformity of genomic coverage was evaluated before CNV calling^18^.

### Validation of the WGS results by conventional methods

#### Copy number analysis of the *PRKAR1A* gene by multiplex-ligation probe amplification reaction (MLPA)

MLPA was performed with probe set SALSA^®^ MLPA^®^ Probemix P481-A1 PRKAR1A-ARMC5 (MRC-Holland, the Netherlands) according to the manufacturer’s recommendations.

#### Breakpoint determination of the large deletion

The maximum length of the deleted gDNA segment was judged based on genome sequencing coverages as well as MLPA results. PCR primers for breakpoint determination were designed in different combinations: deletion-specific primer pairs (Del_For: CAC TAT CTT GCC TAG GCT GGT C and Del_Rev: GGA CAT GAG TAG GGA CTG CAA) flanking the deletion were designed to give product only from the deleted allele under conventional PCR conditions. Normal allele-specific primer pairs (Ref_For: CAC TAT CTT GCC TAG GCT GGT C and Ref_Rev: GGA CAT GAG TAG GGA CTG CAA were designed to give product only from the normal (reference) allele. These primer pairs were applied together in one duplex PCR reaction carried out with Qiagen Multiplex PCR enzyme kit (Hilden, Germany) using the supplier’s recommendations. The reaction was supplemented with 20% Q solution due to the high GC content of the template region. PCR products were run on 1.5% agarose gel electrophoresis and visualized by iBright (Thermo Fisher Scientific, MA, US) gel detection instrument. Additionally, a separate PCR reaction from the mutation carrier gDNA was performed using only the Del_For and Del_Rev primers and the resulting product was bidirectionally Sanger-sequenced on ABI 3030 Genetic Analyzer (Thermo Fisher Scientific, MA, US).

#### Transcript-level analysis, allelic imbalance test

Peripheral blood of the mutation-carrier patient was taken into Tempus blood tube and total RNA was isolated by Tempus Spin RNA Isolation Kit (Thermo Fisher Scientific, MA, US) adhering to the product protocol. First-strand reverse transcription was carried out by ProtoScript II Reverse Transcriptase kit (New England Biolabs, MA, US) both with oligo dT and random hexamer primers. RT-PCR reaction from the cDNA template was performed with exonic primers cDNA_For: CGT CGG TCA GAA AAT GAA GAG T and cDNA_Rev: TTA GAC AGA GCT TGG TGT GAG TTT designed for the detection of an exonic missense variant NM_001276290.1:c.998G > A (rs9789047, MF = 0.17). RT-PCR product was subjected to conventional Sanger sequencing on ABI-3130 Genetic Analyzer Thermo Fisher Scientific, MA, US). The electropherogram peak at the variant position was compared to the same position amplified from gDNA template with primers gDNA_For: TTG GTG TGA GTT TGG GTT TCT and gDNA_Rev: GAA TGG CTG AAG GTT TGG AA.

#### PKA activity assay

For PKA (Protein Kinase A) assay three independent PBMC aliquots of the patient and one aliquot of each control were defrosted, washed two times in 1xPBS and resuspended in 10 ml Gibco PB-MAX Karyotyping medium (Thermo Fisher Scientific, MA, US). Cell suspensions were divided into two parts, 5 ml of each: one was treated with 25 μl 20 mM forskolin (Sigma-Aldrich, Missouri, US) diluted in DMSO (100 μM effective concentration), the other was mock-treated with 25 μl DMSO and both were incubated for 2 h on 37 °C in CO_2_-thermostat. Subsequently, cells were collected and accurate cell number was determined in each sample by cell counting in Neubauer haemocytometer using trypan blue to assign cell viability. PKA assay was carried out employing Invitrogen PKA Colorimetric Activity Kit (Thermo Fisher Scientific, MA, US) according to the manufacturer’s instructions. Lysates from an equivalent number of cells (4 × 10^5^) were tested in serial dilutions from 2 × to 100x. Eight-point standard curve from 50 Unit/ml to 0.3 Unit/ml of the purified enzyme was taken to determine the linear range, where optical density and enzymatic activity are proportional. Colour intensity was defined by plate reader on 450 nm as optical density (OD) numbers. We chose 25x (cell lysate of 6.4 × 10^3^ cells) from the serial dilutions, that best fitted to the linear range of the PKA enzyme standard curve, and the OD figures of this dilution were used for statistical calculations. Two-tailed, unpaired *t* test was applied for determining significance.

## Results

### Identification a 10.66 kbp large deletion in the promoter of the *PRKAR1A* as a pathogenic variant a case with Carney complex

Our proband was first evaluated at our Laboratory in 2010 using gold standard molecular biological methods for testing the suspected gene responsible for the clinical diagnosis. Sanger sequencing of the whole coding region of the *PRKAR1A* gene was performed andno pathogenic variant was detected.

After introducing large scale genetic testing methods in our Laboratory, in 2019, WES was performed in the proband, in one affected and in one clinically unaffected sister. WES did not identify any pathogenic/likely pathogenic variant in the *PRKAR1A* gene and in any gene recommended by ACMG^19^.

**Figure 2:**
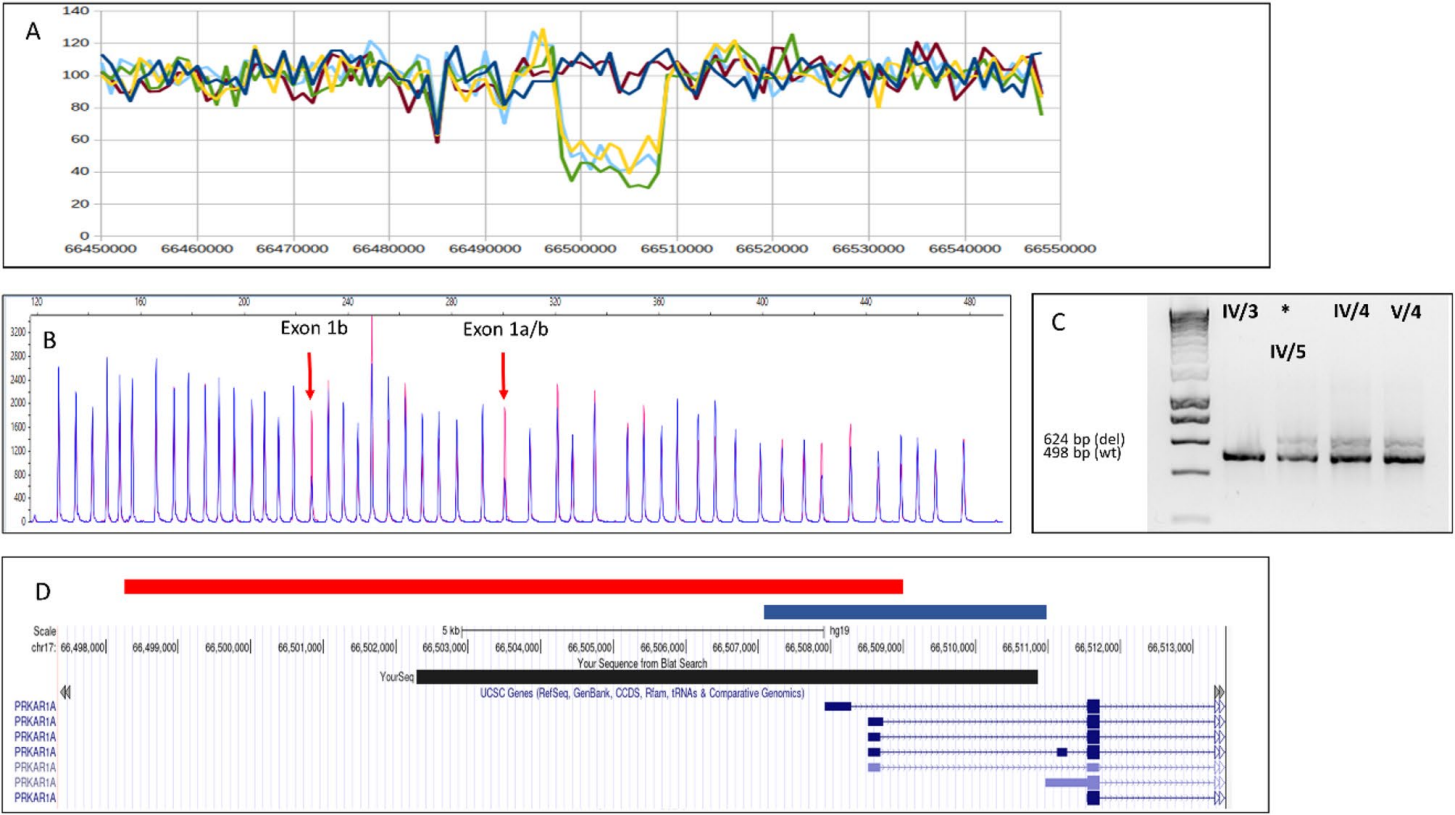
Molecular genetic analysis of the *PRKAR1A* promoter region (**A**) Whole genome sequencing shows half of the mapping reads in affected members compared to controls (x axis: read number, y axis: chromosomal position on chr 17) (**B**) Electropherogram of fragment analysis of the MLPA reaction products analysed by Peak Scanner Software. Reference control sample is coloured in red and *PRKAR1A* deletion carrier is in blue. Half of the peak intensity is detected at the arrow-pointed probes in the deletion carrier sample relative to the control sample. Arrows point to *PRKAR1A* probes specific for Exon 1b and Exon 1a/b. These fragments are falling into the deleted DNA segment. (**C**) Duplex PCR from the gDNA template of the proband (signed with asterisk) and family members (cropped). Deletion-specific product is 624 bp long and obtained only from the gDNA of *PRKAR1A*-deletion carriers with primer pairs Del_For and Del_Rev, while the normal allele-specific product is 498 bp long and produced in each PCR, with primer pairs Ref_For and Ref_Rev if template gDNA has at least one normal allele. Original gels are presented in Supplementary Figure S1. (**D**) Schematic chart of *PRKAR1A* promoter deletions relative to *PRKAR1A* transcripts. The 10.6 kbp deletion detected in our proband is highlighted in red, the 3.88 kbp deletion in blue (Horvath et al.) and the 8.57 kbp deletion (Ito et al.) in black.

Due to the obvious clinical presentation and autosomal heritance we hypothesised that other regions of the *PRKAR1A* gene not analysed by conventional methods or not covered by WES, or other gene might be responsible for the phenotype. Therefore, in 2022, WGS was performed. This method identified a 10.66 kbp large deletion in the promoter of the *PRKAR1A* (Fig. 2a) gene in our proband and first-degree relatives presenting with multiple skin and atrial myxomas. This deletion was absent in the clinically healthy sister of our proband. The identified deletion was further confirmed by MLPA. Probes specific for Exon 1b and Exon 1a/b showed half of the intensity in affected patients compared to healthy control (Fig. 2b). Only these two fragments of the deleted region are included in this commercially available MLPA Kit.

Tumour DNA arising from the cutaneous myxoma of the proband was also tested by MLPA and the same pattern was observed, so the deletion of the gene promoter region was detectable also in the tumour in heterozygous state, and no locus-specific loss of heterozygosity was observed.

### Functional consequences of the detected variant

#### Deletion breakpoint detection

**Figures 3:**
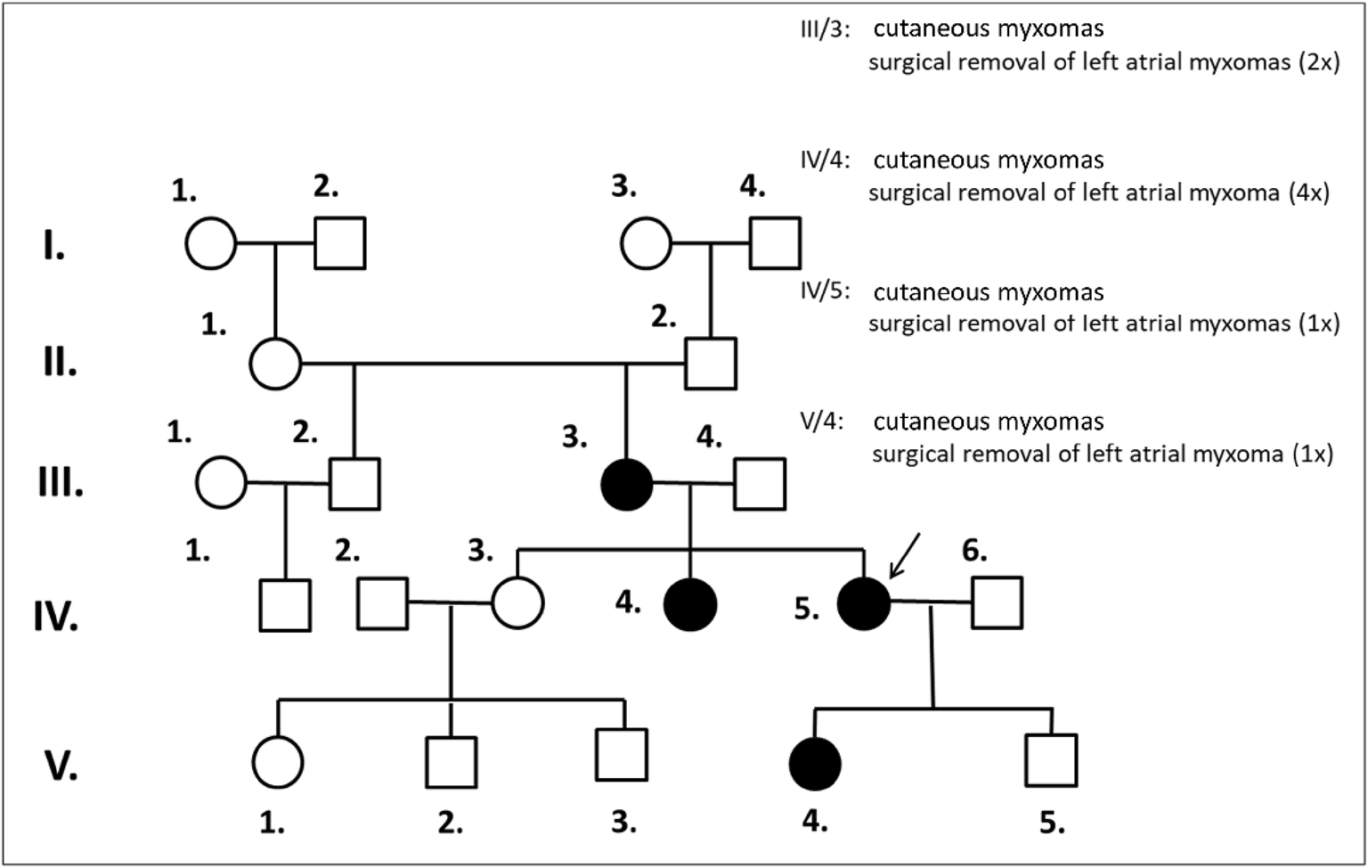
Pedigree chart of family carrying 10.66 kb deletion of the *PRKAR1A* promoter. Black circles indicate family members affected with myxomas. Proband is signed with a black arrow.

Considering the inferred maximum interval of the deletion we designed primers in the immediate flanking genomic region (Del_For and Del_Rev) and performed PCR reaction (see Methods). A PCR product of 624 bp was only obtained from the deleted allele. PCR product was not made from the normal allele since it would be too large to be amplified with conventional amplification parameters. The carrier status was also tested in three first-degree family relatives of the proband. PCR from the deleted allele with primers Del_For and Del_Rev yielded a 624 bp product, whereas PCR from the normal allele with primers Ref_For and Ref_Rev resulted in a 498 bp product and was produced in all PCR reactions, where at least one copy of wild-type allele was present, thus controlling the success of the PCR reaction. The two amplicons of different sizes were easily separated on 1.5% agarose gel (Fig. 2c). Detected patterns on the gel demonstrated that family members IV/4 (the proband’s sister) and V/4 (proband’s daughter) were also mutation carriers, but IV/3 (proband’s sister) did not carry the mutation (Fig. 3). Sequencing of the 624 bp product defined the exact junction point of the deletion and revealed that its size is 10.662 bp. HGVS nomenclature: hg19, chr17:g.66498293_66508954del for the chromosome and LRG_514t1:c.-10403_-7 + 265del relative to the *PRKAR1A* transcript (Fig. 2d). No homology sequence was detectable at the breakpoints.

#### Lack of expression from *PRKAR1A* mutant allele

**Figure 4:**
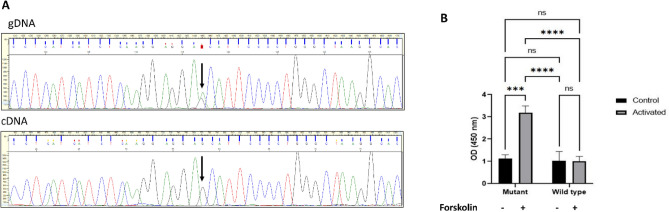
Allelic imbalance test and increased PKA activity upon forskolin treatment in leukocytes harbouring *PRKAR1A* promoter deletion. (**A**) Sequence of exonic heterozygote variant NM_001276290.1:c.998G > A position (pointed out with arrow) and surroundings, amplified from gDNA and cDNA templates respectively of the *PRKAR1A* mutation-carrier proband. Clear 1:1 superposition of G and A nucleotides of the electropherogram is detectable at the variant position of gDNA sequence, whereas no trace of superposition, only the reference G nucleotide is present in cDNA sequence. (**B**) Protein kinase A (PKA) activity test. Optical densities, proportional to PKA enzymatic activities were measured from equivalent PBMC cell lysates (6.4 × 10^3^) of the samples. A ˜threefold increase in PKA activity in the *PRKAR1A* mutation carrier samples (Mutant) compared to wild type was observed upon forskolin stimulation (*p* = 0.0005). Baseline activity without forskolin activation did not yield significant difference between Mutant and Wild Type (*p* = 0.6882).

We suspected that the deletion of the *PRKAR1A* promoter together with the first non-coding exon hinders the binding of transcription factors, therefore restrains transcription. We tested this hypothesis by allelic imbalance experiment, using an exonic heterozygote variant as a marker. WES revealed that gDNA of the deletion-carrier harbours the c.998G > A variant (rs9789047, MAF = 0.17) located in the exon 10 of the *PRKAR1A* gene (NM_001276290.1). The presence of the variant was also confirmed by conventional Sanger sequencing method. The resulting PCR product was sequenced and the electropherogram of the gDNA and cDNA was compared at the variant position (Fig. 4a). No electrophoretic superposition was observed in the cDNA sequence, which indicated monoallelic expression. Since only the reference nucleotide was present at the variant position, we inferred that this variant is in phase (topologically on the same allele) with the deletion and not expressed. To corroborate this conclusion, we sequenced gDNA of this region of other members of the family and yielded that the variant indeed co-segregated with the *PRKAR1A* deletion in each tested case.

#### PKA activity assay

*PRKAR1A* encodes the regulatory subunit (RIα) of the cAMP-dependent protein kinase A (PKA). Reduced quantity of the RIα may elicit overactivity of the PKA enzyme and partially unrestrained PKA-mediated downstream signal transduction pathway. Significant difference was experienced between the deletion carrier compared to control samples upon forskolin activation (two-tailed p-value equals *p* = 0.0006) while no statistically significant difference was observed in basal activity (*p* = 0.68) (Fig. 4b).

## Discussion

In this current study we presented a family where the genetic confirmation of the clinical diagnosis lasted more than 10 years. Our case highlights that in families where the obvious clinical phenotype suggests the presence of a hereditary cancer syndrome but the responsible genetic alteration was not identified by high throughput methods, in our case whole genome sequencing, might be able to finally identify the pathogenic cause. Using WES and WGS for identification of germline pathogenic variants in cases with obvious clinical phenotype where the monogenic syndrome is likely is not recommended. However, as our case demonstrates promoter variants, or deep intronic variants with functional consequences not tested by conventional methods might also be the pathogenic cause for these cases. Therefore, in unresolved cases further studies are required to identify the disease-causing genetic alterations.

The clinical presentation observed in this family was characteristic for Carney complex. Multiple myxomas (cutaneous and atrial) and other dermatological lesions present in three generations were informative for strong genetic background. However, molecular genetic analysis performed with several conventional methods (targeted testing with Sanger sequencing, then WES) failed to identify the underlying genetic cause. In 2022, we had the possibility within the Hungarian Genomic and Bioinformatic Core Facility to perform WGS. This analysis finally revealed the possible pathogenic deletion responsible for the disease. Breakpoint analysis and RNA sequencing clarified that this alteration is 10.6 kb upstream of the *PRKAR1A* gene and includes an active promoter and noncoding exon 1. All affected individuals harbour this alteration while it was absent in the clinically healthy sister of the proband. Pathogenic large deletions of the promoter and noncoding region of the *PRKAR1A* gene are rare alterations. Similarly to our finding, WGS has been successfully applied in the detection of a partial deletion of the *NF1* gene causing neurofibromatosis type 1 even in mosaic form^20^.

To date, only two large deletions involving the promoter region of *PRKAR1A* being responsible for CNC have been identified. The first was reported by Horvath et al. in 2008^21^, while the second by Ito et al. in 2022^22^. All these deletions are partially coincided with the alteration found in our family. Therefore, the pathogenic role of this large 10.6 kb deletion could be also assumed from these studies^21,22^. Our family is the second family (the first was reported by Ito) where no coding exons of the *PRKAR1A* gene were affected by a deletion. Despite the overlapping genomic regions, significant different phenotypes were associated. In the family presented by Ito et al. a bilateral, large‐cell calcified Sertoli-cell testicular tumour and pituitary adenoma in the proband and cardiac myxoma in her mother were observed while in our family the skin lesions dominated the phenotype and only mild endocrine disturbances were detected in the proband and no tumours affecting endocrine organs were observed in deletion carriers.

In CNC, the prevalence of skin symptoms is 80%, the frequent ones being lentiginosis (60–80%), cutaneous myxomas (20–50%) and blue naevi, while less common lesions, such as café-au-lait macules, lipomas, haemangiomas have also been described^23,24^. Lentiginosis is the earliest cutaneous sign and usually precedes the development of endocrine tumours. It is also characteristic for Noonan syndrome with multiple lentigines (NSML) or Peutz-Jeghers-syndrome, however its presence on the lacrimal caruncle, the border of the lips, and eyelid margins is considered the hallmark of CNC. Specific localisations of cutaneous myxomas are the external ear canal, upper eyelids, and areola mammae; however, multiple large myxomas are uncommon^25^. They may be mistaken for neurofibromas, therefore histological examination is always recommended. Cutaneous myxomas can be removed surgically, however, the type of excision should be carefully chosen considering the anatomical region and the size of the lesions. As for genotype–phenotype correlations, c.709-7_709-2del was associated with nonspecific or no dermatological signs^6^, while exonic mutations and c.491-492del were associated with more frequent lentiginosis^5^.

Detailed clinical, hormonal and imaging studies performed in our proband revealed elevated and not suppressible serum cortisol level and increased HOMA index. Imaging did not indicate any lesion present in adrenal regions. The main endocrine feature in CNC is the PPNAD which can be responsible for an ACTH-independent Cushing’s syndrome (CS). Due to the lack of adrenal surgery, we could not prove that PPNAD would be present in our proband. Normal ACTH level, and normal urinary free cortisol (UFC) secretion are against overt hypercortisolism. However, the paradoxical UFC increase on the second day of the high-dose dexamethasone test would be informative for PPNAD. Treatment of patients with CNC is difficult and it is restricted to lesions present in affected individuals. Overt CS due to PPNAD is treated by adrenal surgery, in inoperable cases steroid biosynthesis inhibitors such as ketoconazole, metyrapone or mitotane can be used. Treatment of myxomas is surgical removal of these tumours. Concerning the genetic results, the mild autonomous cortisol secretion and the increased HOMA index metformin treatment was started according to recommendations of the multidisciplinary team within ENDO-ERN.

Metformin improves metabolic profiles of glucocorticoid-treated patients with inflammatory diseases^26^ and has beneficial effects in patients with endogenous GC excess^27^. In addition, metformin is the most widely used oral antihyperglycemic agent, often used in polycystic ovary syndrome and its anticancer effects have been also reported^28^. The action of metformin, however, has not yet been clarified. Activation of AMPK may be the central pathway mediating its interaction with glucocorticoids^29^. After 18-months treatment with a 500 mg daily dose metformin combined with regular physical activity and low-caloric diet a significant improvement in body composition, glucose homeostasis and mood disorder were detected in our proband suggesting the beneficial effects of metformin on all these mild disturbances. The rate of newly developed myxomas also decreased significantly (3 vs. > 10/year) suggesting the antiproliferative effect of metformin.

In summary, our report confirms the relevance of WGS to detect P/LP variants in cases where earlier performed standard molecular genetic testing fails to identify the pathogenic cause. WGS outperformed both WES and targeted testing in our cases which highlights its importance and supports its applicability in clinical practice as a single, large-scale genetic test. WGS might be the choice for molecular analysis of otherwise unresolved cases^30,31^. A novel, large deletion upstream of the *PRKAR1A* gene was proved to be the cause of familiar myxoma which can be considered as Carney complex. The identification of a causal pathogenic variant in our kindred allows adequate genetic counselling and provides access to all those screening and therapeutical approaches which are recommended for patients with CNC.

### Supplementary Information

Supplementary Information.

## Data Availability

We deposited the variant into the LOVD locus specific database, which was already revised and accepted by the curator. The reference number: https://databases.lovd.nl/shared/variants/0000932922
