# Publisher Correction: Whole genome sequencing resolves 10 years diagnostic odyssey in familiar myxoma

**DOI:** 10.1038/s41598-023-43188-6

**Published:** 2023-09-28

**Authors:** Sára Pálla, Judit Tőke, Anikó Bozsik, Henriett Butz, János Papp, István Likó, Enikő Kuroli, András Bánvölgyi, Mátyás Hamar, Jerome Bertherat, Márta Medvecz, Attila Patócs

**Affiliations:** 1 Department of Dermatology, Venereology and Dermatooncology, Semmelweis University, Budapest, Hungary; 2 Department of Internal Medicine and Oncology, Semmelweis University, Budapest, Hungary; 3 ENDO-ERN HCP Semmelweis University, Budapest, Hungary; 4 Department of Molecular Genetics, National Institute of Oncology, Ráth György U. 7-9, 1122 Budapest, Hungary; 5 Hereditary Cancers Research Group, Eötvös Loránd Research Network, Semmelweis University, Budapest, Hungary; 6 National Tumorbiology Laboratory, Budapest, Hungary; 7 Department of Surgery, Transplantation and Gastroenterology, Semmelweis University, Budapest, Hungary; 8 Université Paris Cité, Institut Cochin, Inserm U1016, Paris, France; 9 Department of Endocrinology and National Reference Center for Rare Adrenal Disorders, Hôpital Cochin, Assistance Publique Hôpitaux de Paris, Paris, France; 10 ERN-Skin Semmelweis University, Budapest, Hungary; 11 National Institute of Oncology, Oncology Biobank Center, Budapest, Hungary; 12 Department of Laboratory Medicine, Semmelweis University, Budapest, Hungary; 13 Department of Pathology and Experimental Cancer Research, Semmelweis University, Budapest, Hungary

Correction to: *Scientific Reports* 10.1038/s41598-023-41878-9, published online 05 September 2023

The Data availability section in the original version of this Article was incorrect.

“The datasets generated during and/or analysed during the current study are available from the corresponding author on reasonable request.”

now reads:

“We deposited the variant into the LOVD locus specific database, which was already revised and accepted by the curator. The reference number: https://databases.lovd.nl/shared/variants/0000932922”

The original Article has been corrected.

