# Supplementary figures and images for: Whole genome sequencing resolves 10 years diagnostic odyssey in familiar myxoma
Source: Sci Rep. 2023 Sep 5;13:14658. doi: 10.1038/s41598-023-41878-9 (PMC10480295; doi:10.1038/s41598-023-41878-9)

**Supplementary material**

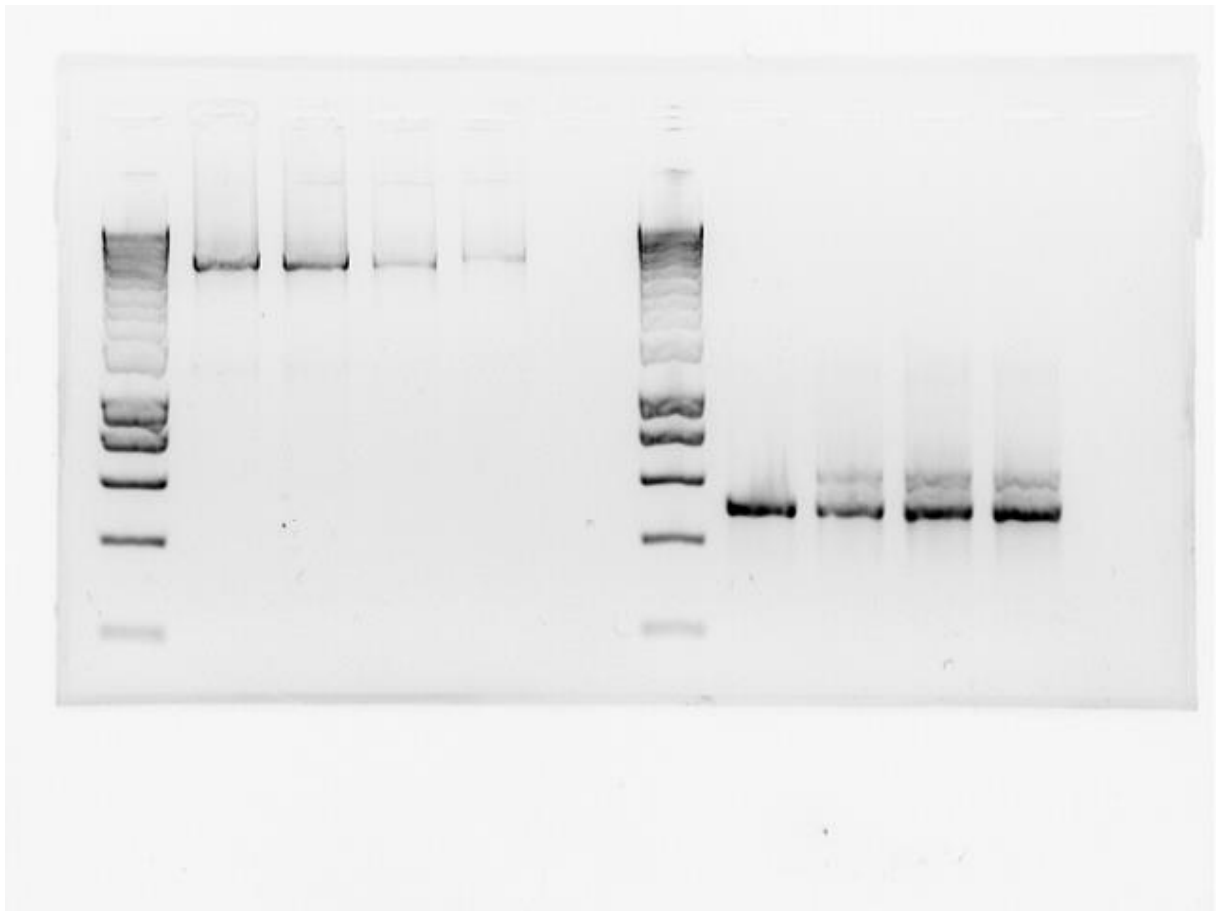

Supplementary Figure S1  
Original image of Figure 2c.

Supplement: Supplementary file 1 — Supplementary Information. [file 41598_2023_41878_MOESM1_ESM.pdf]
